# Fenamate NSAIDs inhibit the NLRP3 inflammasome and protect against Alzheimer's disease in rodent models

**DOI:** 10.1038/ncomms12504

**Published:** 2016-08-11

**Authors:** Michael J. D. Daniels, Jack Rivers-Auty, Tom Schilling, Nicholas G. Spencer, William Watremez, Victoria Fasolino, Sophie J. Booth, Claire S. White, Alex G. Baldwin, Sally Freeman, Raymond Wong, Clare Latta, Shi Yu, Joshua Jackson, Nicolas Fischer, Violette Koziel, Thierry Pillot, James Bagnall, Stuart M. Allan, Pawel Paszek, James Galea, Michael K. Harte, Claudia Eder, Catherine B. Lawrence, David Brough

**Affiliations:** 1 Faculty of Biology, Medicine and Health, University of Manchester, AV Hill Building, Oxford Road, Manchester M13 9PT, UK; 2 St. George's University of London, Institute for Infection and Immunity, Cranmer Terrace, London SW17 0RE, UK; 3 Manchester Pharmacy School, University of Manchester, Manchester M13 9PT, UK; 4 SynAging SAS, 24–30 rue Lionnois, Nancy F-54000, France; 5 Division of Neuroscience, Ninewells Medical School, University of Dundee, Dundee DD1 9SY, UK

## Abstract

Non-steroidal anti-inflammatory drugs (NSAIDs) inhibit cyclooxygenase-1 (COX-1) and COX-2 enzymes. The NLRP3 inflammasome is a multi-protein complex responsible for the processing of the proinflammatory cytokine interleukin-1β and is implicated in many inflammatory diseases. Here we show that several clinically approved and widely used NSAIDs of the fenamate class are effective and selective inhibitors of the NLRP3 inflammasome via inhibition of the volume-regulated anion channel in macrophages, independently of COX enzymes. Flufenamic acid and mefenamic acid are efficacious in NLRP3-dependent rodent models of inflammation in air pouch and peritoneum. We also show therapeutic effects of fenamates using a model of amyloid beta induced memory loss and a transgenic mouse model of Alzheimer's disease. These data suggest that fenamate NSAIDs could be repurposed as NLRP3 inflammasome inhibitors and Alzheimer's disease therapeutics.

## Abstract (web summary)

NSAID-induced analgesia is typically induced by inhibition of COX enzymes. Here the authors show instead that fenamate NSAIDs inhibit the Nlrp3 inflammasome via an effect on volume-regulated anion channel function and also repurpose these drugs for therapeutic effect in rodent models of Alzheimer disease.

## Graphical abstract

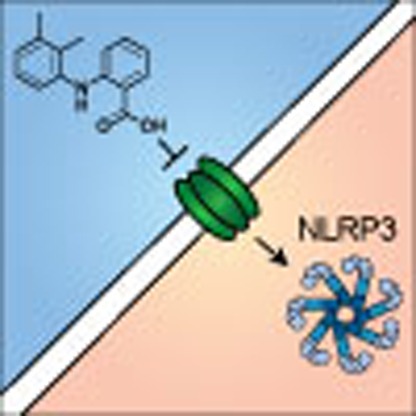

Since their characterization as COX inhibitors^1^, non-steroidal anti-inflammatory drugs (NSAIDs) have been used to treat a wide variety of diseases with relatively limited side effects^2^. NSAID inhibition of COX prevents the conversion of arachidonic acid to eicosanoids resulting in a reduction in the synthesis of proinflammatory prostaglandins^3^. Evidence indicates a polyvalent effect of NSAIDs with some research suggesting COX-independent activity^4^. One such important action may be to directly limit the production of proinflammatory cytokines.

Many inflammatory diseases are driven by the proinflammatory cytokine interleukin-1β (IL-1β)^5^. IL-1β is produced in myeloid cells as an inactive precursor (pro-IL-1β) that requires cleavage by the protease caspase-1 for its activation and secretion^6^. Caspase-1 is also produced as an inactive precursor, which is activated following recruitment to a large multi-protein complex called the inflammasome^6^. Inflammasomes are defined by the presence of a pattern recognition receptor. The best characterized inflammasome-forming pattern recognition receptor, and the most commonly associated with disease is NLRP3 (NLR family, pyrin domain containing 3). In response to pathogen-associated molecular patterns or damage-associated molecular patterns NLRP3 nucleates the oligomerization of ASC (apoptosis-associated speck-like protein containing a caspase recruitment domain) molecules^7^,^8^ into large ‘specks' that serve as platforms for caspase-1 activation and subsequent release of IL-1β.

The NLRP3 inflammasome is an important contributor to inflammatory diseases, including Alzheimer's disease^9^, atherosclerosis^10^, metabolic diseases such as type 2 diabetes^11^ and others^12^. Hence, there is substantial interest in the discovery of potentially therapeutic inflammasome inhibitors. One such compound, MCC950, has been identified as a potent NLRP3-selective inhibitor^13^, but is not yet available for clinical use. Fenamate NSAIDs have been shown to inhibit IL-1β secretion from macrophages^14^, although the significance of COX inhibition remains unclear^15^. Here we show that the fenamate class of NSAIDs inhibit the NLRP3 inflammasome via reversible blockade of volume-regulated anion channels (VRAC) in the plasma membrane, and inhibit cognitive impairments in models of Alzheimer's disease in rodents, thus offering a safe and rapidly translatable option to treat NLRP3-related inflammatory diseases.

## Results

### Fenamate NSAIDs selectively inhibit the NLRP3 inflammasome

**Figure 1:**
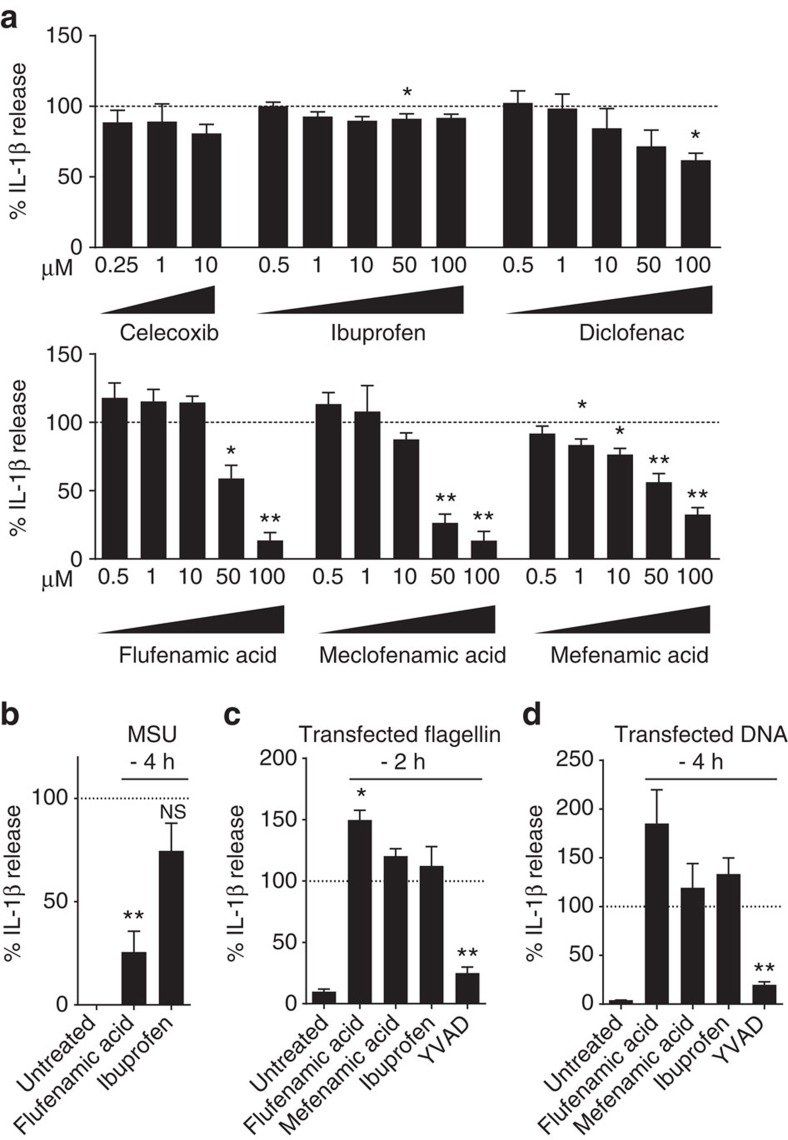
Fenamate NSAIDs inhibit IL-1β processing and release. (**a**) iBMDMs were primed with LPS (1 μg ml^−1^, 2 h) then pre-treated with NSAID at indicated concentration before stimulating with ATP (5 mM, 1 h). (**b**–**d**) Murine primary BMDMs from WT (**b**) or NLRP3^−/−^ (**c**,**d**) mice were primed with LPS (1 μg ml^−1^, 4 h) and pre-treated with NSAID (100 μM, 15 min) before stimulating with monosodium urate (MSU) crystals (250 μg ml^−1^, 4 h) (**b**), transfected ultrapure flagellin from *Salmonella typhimurium* (1 ng per 1,000 cells, 2 h) (**c**), or transfected DNA (0.66 ng per 1,000 cells, 4 h) (**d**). Supernatants were analysed by ELISA. Data are presented as mean % IL-1β release versus vehicle (DMSO) control+s.e.m (*n*=3 or 4). NS, not significantly different, *P<0.05, **P<0.01 determined by one-sample *t*-test versus hypothetical value of 100%.

Immortalized mouse bone marrow-derived macrophages (iBMDMs) were primed with lipopolysaccharide (LPS; 1 μg ml^−1^, 2 h), after which the media was replaced with serum-free media. At this point the cells were incubated with a range of NSAIDs (Supplementary Fig. 1) for 15 min before 1 h stimulation with 5 mM ATP to activate the P2X7 receptor and induce NLRP3 inflammasome activation^16^. ELISA analysis of cell supernatants revealed that of the NSAIDs tested the fenamates (N-phenyl-substituted anthranilic acid derivatives such as flufenamic acid, meclofenamic acid, mefenamic acid) were most effective at inhibiting IL-1β release (Fig. 1a). The selective COX-2 inhibitor celecoxib did not inhibit IL-1β release, nor did ibuprofen, even at concentrations supra-maximal for COX inhibition. Western blot analysis of the supernatants also showed that caspase-1-dependent processing of IL-1β was also inhibited by the fenamates (Supplementary Fig. 2). Fenamate NSAIDs had no effect on ATP-induced cell death (Supplementary Fig. 3) suggesting that their effects were specific to IL-1β release, and independent of the stability of ATP. As early studies indicated multiple sites of action for the fenamate NSAIDs^17^, these data reveal the fenamates as inhibitors of IL-1β processing and release and suggest that this effect is independent of COX inhibition.

Flufenamic acid, but not ibuprofen, also inhibited IL-1β release induced by the NLRP3 inflammasome activator monosodium urate (MSU) in primary mouse BMDMs (Fig. 1b), suggesting that their effect was not due to direct inhibition of the P2X7 receptor or a direct effect on ATP. To determine whether the fenamates were selective inhibitors of NLRP3-dependent IL-1β release, we tested their effects against other well-characterized inflammasomes in primary BMDMs. NLRC4 inflammasome activation by transfected *Salmonella typhimurium* flagellin in BMDMs induced IL-1β release, which was not inhibited by flufenamic or mefenamic acid (Fig. 1c). AIM2 inflammasome activation by transfected double-stranded DNA was also unaffected by flufenamic or mefenamic acid (Fig. 1d). These data suggest that the fenamates selectively inhibit the NLRP3 pathway.

**Figure 2:**
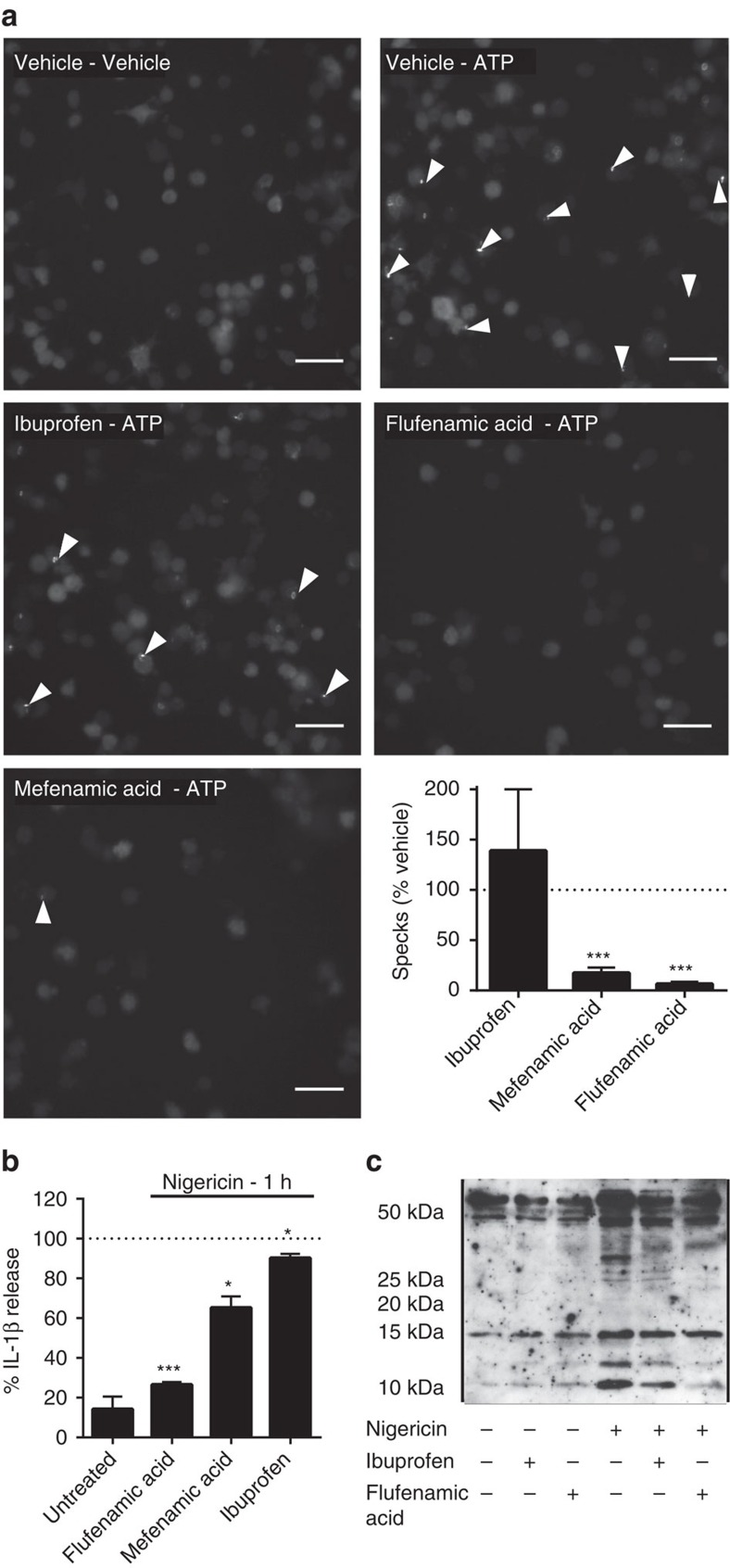
Fenamate NSAIDs inhibit ASC speck formation and caspase-1 activation. (**a**) iBMDMs stably expressing ASC protein conjugated to mCherry were primed with LPS (1 μg ml^−1^, 2 h) then pre-treated with selected drug (100 μM, 15 min) before stimulation with ATP (5 mM, 30 min) under live microscopy. Formation of ASC specks (examples indicated by white arrows) was quantified (lower right) and presented as mean % specks counted versus vehicle+s.e.m (*n*=4). Scale bars are 20 μm. (**b**,**c**) THP-1 cells were primed with LPS (1 μg ml^−1^, 4 h) and pre-treated with NSAID (200 μM, 15 min) before stimulating with nigericin (10 μM, 1 h). Supernatants were taken and analysed for IL-1β by ELISA (**b**) and the p10 active subunit of caspase-1 by western blot (**c**). ELISA data are presented as mean % IL-1β release versus vehicle (DMSO) control+s.e.m (*n*=3). *P<0.05, ***P<0.001 determined by one-sample *t*-test versus hypothetical value of 100%.

iBMDMs were transduced with lentivirus to stably express the inflammasome adaptor ASC fused to mCherry. Inflammasome activation in these cells results in the aggregation of ASC into one large ASC speck in the cytoplasm that is readily visible by fluorescence microscopy. ASC–mCherry expressing iBMDMs were treated with LPS (1 μg ml^−1^, 2 h) and then ATP (5 mM) in serum-free media plus or minus a 15 min pre-incubation with ibuprofen, mefenamic acid or flufenamic acid (all 100 μM). Flufenamic and mefenamic acid both inhibited NLRP3-dependent ASC speck formation whereas ibuprofen had no effect (Fig. 2a). In addition to inhibiting IL-1β release from mouse cells, flufenamic acid also inhibited IL-1β release (Fig. 2b) and production of the active p10 subunit of caspase-1 (Fig. 2c) from nigericin-treated human THP-1 cells. These data show that the fenamates inhibited NLRP3-dependent IL-1β release upstream of ASC speck formation.

### Chloride channels regulate the effects of fenamates on NLRP3

**Figure 3:**
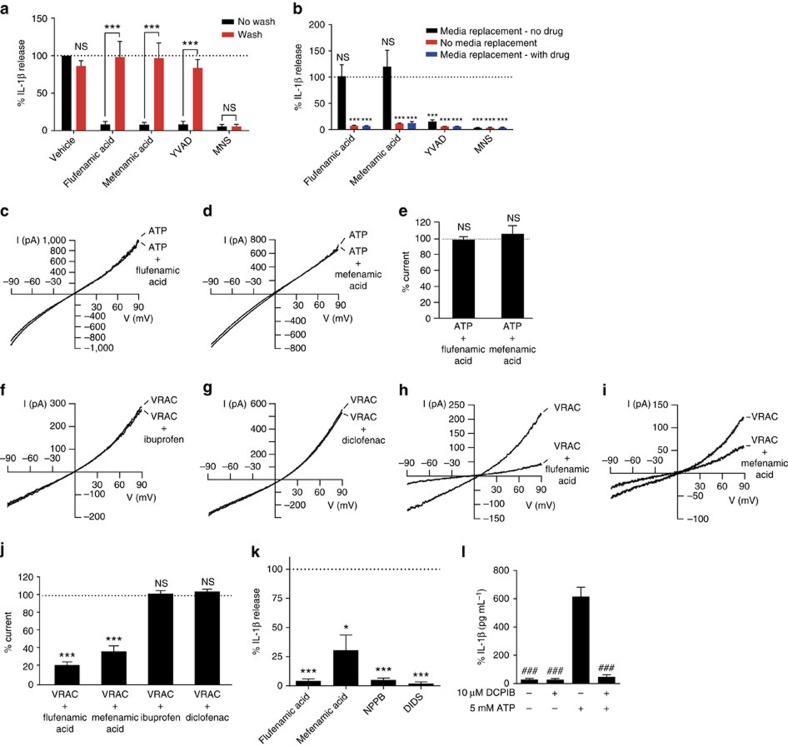
Fenamates inhibit NLRP3 via reversible blockade of the Cl^−^ channel VRAC. (**a**) iBMDMs were primed with LPS (1 μg ml^−1^, 4 h) and pre-treated with NSAID, reversible caspase-1 inhibitor YVAD-CHO or irreversible NLRP3 inhibitor 3,4-methylenedioxy-β-nitrostyrene (MNS) (100 μM, 15 min) before washing 3 × 5 min (or no wash) and stimulation with ATP (5 mM, 1 h). (**b**) Cells were pre-treated with drug as above before either media replacement without drug, media replacement with drug or no media replacement followed by ATP stimulation. (**c**–**e**) iBMDMs were primed as above. Currents were measured in the whole-cell configuration of the patch clamp technique. Voltage ramps were applied from −90 mV to +90 mV for a duration of 360 ms every 20 s. Non selective cation currents were evoked by 5 mM ATP and were measured in the absence (ATP) or presence of 100 μM flufenamic acid (**c**, *n*=8) or mefenamic acid (**d**, *n*=10). (**f**–**j**) iBMDMs were primed as above before whole-cell volume-regulated chloride currents (VRAC) were induced by hypotonic extracellular medium. Currents were induced by 360 ms-lasting voltage ramps from −90 mV to +90 mV every 20 s. Examples of current recordings measured in the absence or presence of 100 μM ibuprofen (**f**, *n*=6), diclofenac (**g**, *n*=5), flufenamic acid (**h**, *n*=6), or mefenamic acid (**i**, *n*=6). ***P<0.001, NS, not significantly different, determined by one-sample *t*-test versus hypothetical value of 100%. (K) iBMDMs were primed as above and pre-treated with flufenamic acid, mefenamic acid, Cl^−^ channel blockers NPPB or DIDS (100 μM, 15 min) before stimulation with ATP (5 mM, 1 h). (**l**) iBMDMs were primed as above and pre-treated with DCPIB (10 μM, 15 min) before stimulation with ATP (5 mM, 1 h). IL-1β in the supernatants (**a**,**b**,**k**,**l**) was quantified by ELISA and data are presented as mean % IL-1β release versus vehicle (DMSO) control+s.e.m or mean IL-1β release (*n*=4). *P<0.05, **P<0.01, ***P<0.001, NS, not significantly different determined by one-way ANOVA with Tukey's *post hoc* analysis (**a**) or one-sample *t*-test versus hypothetical value of 100% (**b**–**k**). ^###^*P*<0.001 versus ATP (**l**).

To further identify the mechanism of action of fenamates on NLRP3, we sought to determine the reversibility of fenamate inhibition of NLRP3-dependent IL-1β release. Flufenamic acid did not react with protected cysteine in contrast to 3,4-methylenedioxy-β-nitrostyrene (MNS; Supplementary Fig. 4), a known inhibitor of NLRP3 via irreversible cysteine modification^18^,^19^. Thus the mechanism of fenamate action does not involve irreversible cysteine modification. Following the secretion protocol above, except with three media changes over 15 min to wash out the fenamates flufenamic acid and mefenamic acid, the reversible caspase-1 inhibitor Ac-YVAD-cho, and the irreversible NLRP3 inhibitor MNS, it was revealed that the inhibitory effects of the fenamates on NLRP3-dependent IL-1β release were fully reversible (Fig. 3a). A further experiment, with just one wash, which failed to remove the inhibitor effects of YVAD, completely reversed the effects of flufenamic and mefenamic acid on NLRP3 inflammasome inhibition (Fig. 3b) strongly suggesting that their inhibitory effects were at the plasma membrane. Flufenamic acid is a well-established modulator of ion channels^20^. Thus we first-tested the effects of the flufenamic and mefenamic acid on ATP-induced cation currents in LPS-primed iBMDMs using whole-cell patch clamp recordings. Neither flufenamic nor mefenamic acid inhibited ATP-induced cation currents (Fig. 3c–e), nor inhibited ATP-induced increases in intracellular Ca^2+^ or Na^+^ levels (Supplementary Fig. 5). Flufenamic acid is also known to inhibit Cl^−^ currents^20^ and so we tested the effects of the fenamates on VRAC. VRAC currents measured by whole-cell patch clamp in LPS (1 μg ml^−1^, 4 h) primed iBMDMs were induced by hypotonicity, also a known activator of the NLRP3 inflammasome^21^. Both flufenamic and mefenamic acid inhibited VRAC, while non-NLRP3 inhibiting NSAIDs such as ibuprofen and diclofenac had no effect (Fig. 3f–j). In light of these data we tested whether other Cl^−^ channel inhibitors would block NLRP3-dependent IL-1β release. In LPS-primed iBMDMs, a 15 min pre-incubation with either of the Cl^−^ channel inhibitors NPPB (100 μM) or 4,4′-diisothiocyanatostilbene-2,2′-disulfonic acid disodium salt hydrate (DIDS; 100 μM) completely prevented NLRP3-inflammasome-dependent IL-1β release (Fig. 3k). To further confirm the importance of Cl^−^ currents, and in particular the importance of VRAC, we used the specific VRAC inhibitor DCPIB (10 μM), which also completely inhibited ATP-induced IL-1β release (Fig. 3l). Flufenamic acid and DCPIB also inhibited the regulatory volume decrease induced by hypotonicity in THP-1 cells, which is known to require Cl^−^ efflux^22^ (Supplementary Fig. 6). These data suggest that the mechanism of action of the fenamate NSAIDs on the NLRP3 inflammasome is via an inhibition of the Cl^−^ channel VRAC.

### Fenamates inhibit NLRP3 inflammasomes *in vivo*

We next tested whether the fenamates would have any effect on the NLRP3 inflammasome *in vivo*. Initially, we used a mouse air pouch model in which MSU-induced IL-1β production is NLRP3 inflammasome dependent^23^. Seven days after the air pouches were raised in the dorsum of C57BL/6 mice they were injected with MSU crystals (3 mg in 1 ml sterile saline) or vehicle with or without flufenamic acid (20 mg kg^−1^ in sterile saline, 5% Cremaphor EL, 5% dimethyl sulfoxide (DMSO)) or vehicle. Six hours later pouches were lavaged and IL-1β measured by ELISA. Flufenamic acid significantly inhibited MSU-induced IL-1β production in the pouch (Supplementary Fig. 7). Flow cytometry was performed on lavage samples collected from the air pouch and a specific MSU-dependent recruitment of macrophages/monocytes was observed (Supplementary Fig. 7). Flufenamic acid also inhibited MSU-induced macrophage/monocyte recruitment (Supplementary Fig. 7). We also tested mefenamic acid in a NLRP3-dependent peritoneal model of inflammation. LPS injection into the peritoneum induces low levels of IL-1β that are greatly enhanced with the co-addition of the NLRP3 agonist ATP^24^,^25^,^26^. In this model the IL-1β production is completely dependent on P2X7 (ref. 25), an established activator of the canonical NLRP3 inflammasome^16^, and is inhibited by cytokine release inhibiting drugs developed by Pfizer^26^, which are close analogues and precursors of the NLRP3-selective inflammasome inhibitor MCC950 (ref. 13). Wild-type and NLRP3^−/−^ mice were treated with LPS (1 μg in 0.5 ml, intraperitoneal (i.p.)) for 4 h followed by ATP (100 mM in 0.5 ml) for 15 min. Groups of wild-type mice were also dosed with mefenamic acid, or MCC950 (both at 50 mg kg^−1^, i.p.). Peritoneums were lavaged, blood taken and lavage fluid and plasma were analysed by ELISA for IL-1β and IL-1α (Supplementary Fig. 8). Peritoneal administration of LPS and ATP induced significant release of IL-1β that was completely blocked by mefenamic acid and by MCC950 (Supplementary Fig. 8). Furthermore, LPS and ATP did not cause IL-1β release from NLRP3^−/−^ mice (Supplementary Fig. 8). These data suggest that fenamates are effective inhibitors of the NLRP3 inflammasome *in vivo*.

**Figure 4:**
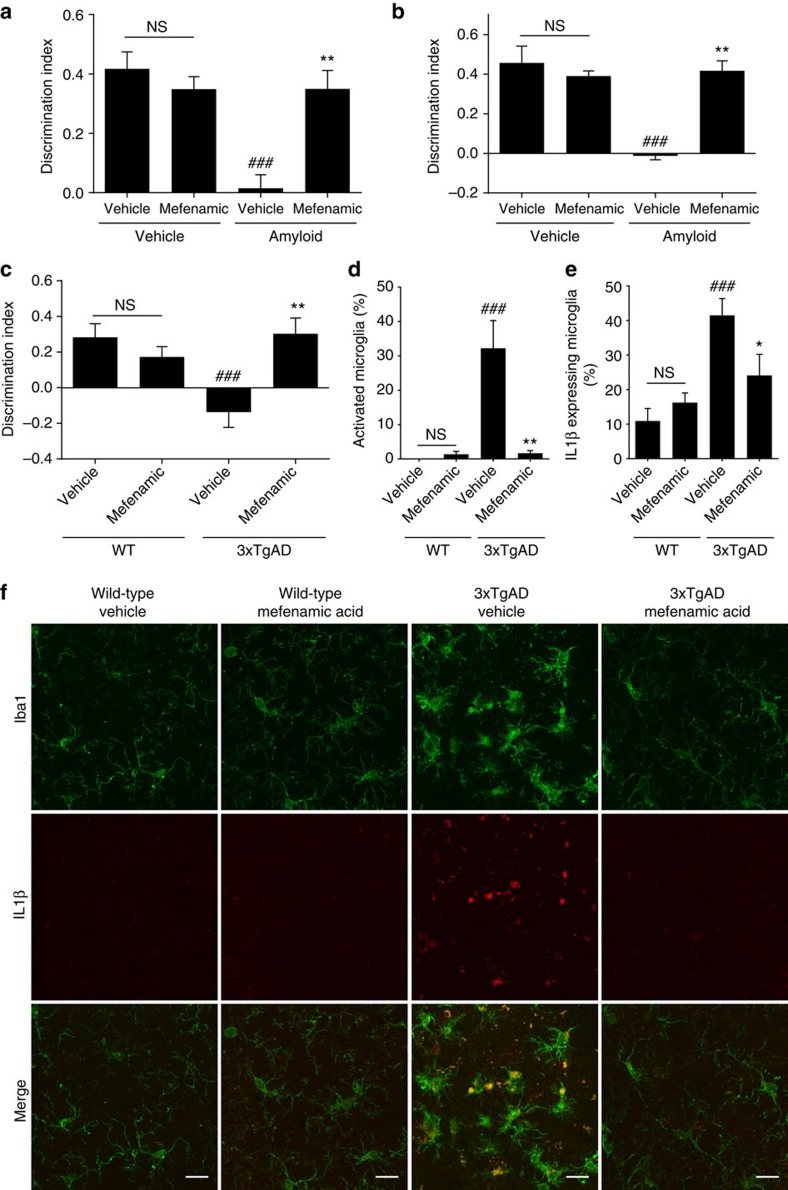
Mefenamic acid is protective in rodent models of Alzheimer's disease through an anti-inflammatory mechanism. (**a**,**b**) Female Lister hooded rats (200–230 g) received an acute unilateral intracerebroventricular injection of soluble Aβ_1–42_ on day 0 (5 nmol in 10 μl) which was followed by 14 days (starting one day before surgery) of i.p. injection of mefenamic acid (5 mg kg^−1^) or vehicle. Animals were then tested in the novel object recognition task on 14 d (**a**) and 35 d (**b**) post surgery. Discrimination index data are presented as mean+s.e.m (*n*=5–10). NS, not significantly different, ^###^*P*<0.001 compared with vehicle/vehicle treated animals and **P<0.01 compared to Aβ1-42/vehicle group. (**c**–**f**) 13–14 month old transgenic mouse model of Alzheimer's 3 × TgAD and wild-type (WT) control were treated with vehicle or 25 mg kg^−1^ day^−1^ mefenamic acid. (**c**) On day 18 memory was assessed with the novel object recognition task; discrimination index data are presented as mean+s.e.m (*n*=8–10). ^###^*P*<0.001 compared to vehicle/WT animals and **P<0.01 compared with vehicle/3 × TgAD mice. (**d**–**f**) Evaluation of Iba1 and IL-1β expressing microglia within the subicula of 3xTgAD and WT mice following vehicle or mefenamic acid treatment. Microglial activation (**d**) and IL-1β expression (**e**) were evaluated and presented as mean+s.e.m (*n*=8–10). ^###^*P*<0.001 compared with vehicle/WT animals and **P<0.01 compared to vehicle/3 × TgAD mice. (**f**) Representative images of microglial activation and IL-1β co-localization of 3 × TgAD and WT subicula following vehicle or mefenamic acid treatment. Scale bars are 15 μm. Statistical analyses performed using two-way ANOVA followed by Sidak corrected *post hoc* analysis.

We also tested the effect of fenamates in more disease relevant models. Inflammation contributes to the progression of Alzheimer's disease^27^, and on-going research is establishing the NLRP3-inflammasome as central to the development of inflammation, pathology and memory deficits in a mouse model of Alzheimer's disease^9^,^28^. Amyloid beta (Aβ) is a causative factor in the development of Alzheimer's disease and is a known activator of the NLRP3 inflammasome^28^. Mefenamic acid is used routinely in clinical practice and so we tested the effects of mefenamic acid in an *in vivo* model of Aβ-induced memory loss^29^. Rats injected intracerebroventricularly with soluble Aβ_1–42_ oligomers or vehicle were also treated with daily injections of mefenamic acid (5 mg kg^−1^, i.p.) or vehicle for 14 days. Mefenamic acid treatment prevented Aβ_1–42_ induced memory deficits as measured using the novel object recognition (NOR) test at day 14 (Fig. 4a, Supplementary Fig. 9). This protective effect was sustained up to day 35, 21 days after the mefenamic acid treatment had been terminated (Fig. 4b).

Following the acute Aβ_1–42_ injection model, we then tested the efficacy of mefenamic acid in 3 × TgAD transgenic mice (a mouse model for Alzheimer's disease). For this we used a therapeutic administration strategy beginning at an age where pathology and memory deficits have previously been described in the 3 × TgAD model^30^. At 13–14 months of age administration of mefenamic acid by osmotic minipump over 28 days (at 25 mg kg^−1^ per day) completely abated memory deficits in the 3 × TgAD mice as measured by NOR (Fig. 4c). The brains of these mice were sectioned and stained for IL-1β and, using Iba1, for microglia. Microglial activation states were scored by morphology as described in the Methods section (Supplementary Fig. 10, modified scale from published literature^31^,^32^). In 3 × TgAD mice it has previously been established that amyloid pathology begins to develop in the subiculum at around 12 months of age^30^. We found that there was substantial microglial activation in the subicula of 3 × TgAD mice, and that the activated/amoeboid microglia expressed IL-1β (Fig. 4d–f, Supplementary Fig. 10). No microglial activation or IL-1β expression was seen in other regions of the brain (Supplementary Fig. 11). Mefenamic acid treatment completely abated Alzheimer's disease-related neuroinflammation with levels of microglial activation and IL-1β expression reduced to that of wild-type mice (Fig. 4d–f, Supplementary Fig. 10).

## Discussion

The NLRP3 inflammasome is an important contributor to diverse inflammatory diseases including Alzheimer's disease^9^, atherosclerosis^10^, metabolic diseases such as type II diabetes^11^, and others^12^. However, despite this, there are currently no clinically available inhibitors of NLRP3. Here we show that fenamate NSAIDs are selective inhibitors of the NLRP3 inflammasome. We also show that the fenamates inhibited the membrane Cl^−^ channel VRAC. Given additional recent research showing the importance of Cl^−^ channels in inflammasome activation in response to hypotonicity^21^, we propose that the effects of fenamates on NLRP3 are *via* inhibition of VRAC. Furthermore we show that inhibiting this pathway was protective and therapeutic in two animal models of Alzheimer's disease. Treatment with mefenamic acid of an established disease model abated brain inflammation and memory deficits suggesting that inflammation is a druggable target for Alzheimer's disease.

There has been a significant decline in the number of new therapies being translated to clinic in the last two decades^33^. However, we now appreciate that the robustness of biological systems depends on the network and there is an appreciation that targeting multiple points in a pathway may be more efficacious than targeting a single node^33^,^34^. The fenamates inhibit COX enzymes^35^, and as we report here, also inhibit the NLRP3 inflammasome. Therefore the fenamate NSAIDs are attractive candidates for a polyvalent approach to treat inflammatory disease. The mechanism of action of the fenamates *in vivo* may be as much dependent on VRAC/NLRP3 as it is COX. We have previously reported synergy between inhibitors in inflammatory pathways to cause levels of inhibition far greater than observed singly^34^. Thus the ability of molecules such as the fenamates to modulate several points in a pathway is likely to confer greater protective effects than molecules that have single targets, thus allowing them to be effective at lower doses.

The selectivity of the fenamates to NLRP3 over other inflammasomes reported here is another advantage since their use would avoid compromising NLRC4 or AIM2 inflammasome-dependent host responses to infection. The current strategies used to target IL-1β in disease rely on biologicals such as the IL-1 receptor antagonist anakinra, or the neutralizing antibody canakinumab^36^. While these are effective in some inflammatory diseases, they are expensive and may not readily penetrate tissues such as the brain. Thus, used as a monotherapy, or as an adjunct to current therapies, there is real scope to consider fenamate NSAIDs as a frontline treatment for inflammatory disease.

Although there is robust evidence linking NLRP3 to inflammatory disease from animal models (cited above), apart from the cryopyrin-associated periodic syndromes, there is limited evidence in humans. Further characterization of human inflammatory disorders may widen the scope for use of fenamate NSAIDs to target NLRP3 in human disease. Given that some fenamate NSAIDs are already routinely used clinically, and their pharmacokinetic and toxicity profiles are well established^37^ encouraging results in animal studies could lead rapidly to clinical efficacy trials in inflammatory diseases. Additional epidemiological studies to assess benefits to patients taking fenamate NSAIDs in NLRP3/IL-1β-associated diseases would also inform and facilitate their future use.

For almost 30 years VRAC has been studied^38^. However, it is only recently that the molecular identity of VRAC was discovered, when two studies revealed that leucine rich repeat containing 8 A (LRRC8A) is an essential component^39^,^40^. LRRC8A assembles channels with other LRRC8 proteins to reconstitute the hypotonic-activated VRAC conductance^39^,^40^. These studies, and our own data presented here, highlight VRAC as a potential drug target in inflammatory disease, opening the door for the development of new and improved molecular and pharmacological tools to further dissect the importance of VRAC in the regulation of the NLRP3 inflammasome and for the development of new therapeutic strategies.

In summary, we have shown that fenamate NSAIDs are potent and selective inhibitors of the NLRP3 inflammasome, which act through inhibition of VRAC, a novel player in NLRP3 inflammasome regulation. Fenamate NSAID inhibition of NLRP3 is rapidly reversible which offers significant clinical benefit. We have characterized their activity both *in vitro* and *in vivo* and propose that fenamate NSAIDs can be rapidly repurposed as drugs to target the NLRP3 inflammasome in inflammatory diseases such as Alzheimer's disease.

## Methods

### Materials

Pharmacological reagents were obtained from the following manufacturers: Sigma (celecoxib, ibuprofen, diclofenac, flufenamic acid, meclofenamic acid, mefenamic acid, 5-nitro-2-(3-phenylpropylamino)benzoic acid (NPPB), DIDS, 4-(2-Butyl-6,7-dichlor-2-cyclopentyl-indan-1-on-5-yl) oxybutyric acid (DCPIB), Lipopolysaccharide from *Escherichia coli* O26:B6, ATP and nigericin), Merck-Millipore (Ac-YVAD-CHO), Acros organics (MNS) and Invivogen (MSU crystals, ultrapure flagellin from *Salmonella typhimurium*). All other materials/reagents were obtained from Sigma unless otherwise stated.

### Cell culture and assays

iBMDMs^41^ were obtained from Clare Bryant (Department of Veterinary Medicine, University of Cambridge). iBMDMs were cultured in Dulbecco's Modified Eagle's Medium (DMEM), 10% fetal bovine serum (FBS, Life Technologies), 100 U ml^−1^ penicillin and 100 μg ml^−1^ streptomycin (PenStrep). Cells were seeded overnight at 5 × 10^5^ ml^−1^ in 24- or 96-well plates. Cells were primed with LPS then treated with drug or vehicle (DMSO) in serum-free media for 15 min. Following drug incubation, inflammasomes were stimulated by adding ATP (5 mM) for 1 h. Supernatants were removed and analysed for IL-1β content by ELISA (DuoSet, R&D systems) according to manufacturer's instructions. Primary mouse BMDMs were isolated by flushing marrow from femurs and tibias of wild-type C57BL/6 (Charles River) or NLRP3^−/−^ mice and cultured with L929 mouse fibroblast supernatant-conditioned media for 7–10 days. Human THP-1 peripheral blood monocyte-like cells were cultured in RPMI-1640 Medium supplemented with 10% FBS, 100 U ml^−1^, 100 μg ml^−1^ PenStrep, 20 mM l-Glutamine and 55 μM 2-mercaptoethanol (Life Technologies). Following priming for 4 h with LPS and 15 min pre-treatment with drug in serum-free media, inflammasomes were activated with nigericin (10 μM) for 1 h, MSU crystals (250 μg ml^−1^) for 4 h, transfected ultrapure flagellin from *Salmonella typhimurium* (100 ng per 100,000 cells) for 2 h, or transfected DNA (pEF/V5-His A plasmid empty vector (Life Technologies, 66.7 ng per 100 K cells)) for 4 h. Transfections described above were performed using Lipofectamine 3000 (Life Technologies) according to manufacturer's instructions.

### Western blotting

Western blot analysis was performed on supernatants for IL-1β and caspase-1. Samples were run on 12% (IL-1β) or 15% (caspase-1) SDS polyacrylamide gels. For caspase-1 blots THP-1 supernatants were concentrated before preparation and loading by centrifugal filtering (Amicon 10 K centrifugal filters) according to manufacturer's instructions. Gels were transferred at 15 V onto nitrocellulose membrane (GE Life Sciences) using a Trans-Blot s.d. semi-dry transfer system (Bio-Rad) before blocking with 5% w/v milk in phosphate-buffered saline, 1% Tween 20 (Sigma) (PBST) for 1 h at room temperature. Membranes were washed and incubated (4 °C) overnight in goat anti-mouse IL-1β (100 ng ml^−1^, R&D Systems) or rabbit anti-human caspase-1 p10 (666.6 ng ml^−1^, Santa Cruz) primary antibodies in PBST, 0.1% bovine serum albumin (BSA). Following this, membranes were washed and incubated with rabbit anti-goat (550 ng ml^−1^) or goat anti-rabbit IgG (250 μg ml^−1^, Dako) in 5% milk in PBST for 1 h at room temperature. Finally, membranes were washed and incubated in Amersham ECL Western Blotting Detection Reagent (GE Life Sciences) before exposure on photographic film (Scientific Laboratory Supplies). Uncropped blots are presented in Supplementary Fig. 12.

### Cell death assays

Cell death was measured by assessing lactate dehydrogenase release using the CytoTox 96 Non-Radioactive Cytotoxicity Assay (Promega) according to manufacturer's instructions.

### ASC speck imaging

Live imaging of ASC speck formation was performed using iBMDMs transfected to stably express ASC conjugated to mCherry protein. Mouse ASC (Accession Number NM_023258) was amplified by PCR using primers flanked by gateway recombination sequences (forward primer: 5′- ATGGGGCGGGCACGAGATGCC -3′, reverse primer: 5′- GCTCTGCTCCAGGTCCATCAC -3′). A third generation ‘pLNT' lentiviral transfer system was used to express N-terminally mCherry-tagged amplified *ASC* gene from a constitutive ubiquitin-ligase C promoter^42^. Stably transduced cells were plated overnight at 5 × 10^5^ cells per ml. The following day, cells were primed with LPS (1 μg ml^−1^, 2 h). 1 h into priming, Hoechst 33342 (2 μg ml^−1^, Immunochemistry) was added to aid identification of the cells. Following priming, media was changed to DMEM containing 25 mM HEPES pH 7.4 and cells transferred to a BD Pathway Bioimager 855 (BD Biosciences) and imaged at 37 °C. Cells were pre-treated with drug or vehicle for 15 min before imaging. Images were acquired using a × 20/0.75 UApo objective and the following filter setups: Hoechst Ex. 360/10 Di. Em. 84101; mCherry Ex. 555/28 Di. Em. 84101. Images were collected and a montage of 3 × 3 was created. Following drug incubation, cells were stimulated by addition of ATP (5 mM) directly into the well and imaged as described for 30 min. Specks were quantified by blind-counting nine separate fields of view (1.2 mm^2^) using ImageJ.

### Cysteine modification and NMR

Flufenamic acid or MNS (0.05 mmol) was dissolved in DMSO-*d*_*6*_ (400 μl) in an NMR tube. *N*-Acetyl-L-cysteine methyl ester (17.7 mg, 0.10 mmol) solubilized in DMSO-*d*_*6*_ (100 μl) was added and an NMR spectrum was recorded every 10 min after the addition for 2 h and then every 1 h until 24 h. A Bruker Avance 400 spectrometer was used to record ^1^H spectra at 300.1 MHz. Chemical shifts are defined in parts per million and referenced against tetramethylsilane (*δ*=0). NMR analysis was conducted using Bruker's TopSpin software package.

### Electrophysiological recordings

Membrane currents were measured using the whole-cell configuration of the patch-clamp technique. An EPC-10 patch-clamp amplifier (HEKA) was interfaced to a computer for pulse application and data recording using the programme PatchMaster (HEKA). Patch electrodes of 3-5 MΩ were fabricated on a two-stage puller (Narishige PC-10) from borosilicate glass (Hilgenberg). For all electrophysiological experiments, 10^5^ iBMDM cells were seeded in 24-well plates on glass coverslips and primed the next day with 1 μg ml^−1^ LPS for 4 h. For electrophysiological recordings of ATP-induced cation currents, patch electrodes were filled with an intracellular solution containing (in mM): KCl, 120; HEPES, 10; EGTA, 11; CaCl_2_, 1; MgCl_2_, 2 (pH 7.3). LPS-primed iBMDM cells were superfused with extracellular solution containing (in mM): NaCl, 130; KCl, 5; HEPES, 10; D-glucose, 10; CaCl_2_, 2; MgCl_2_, 1 (pH 7.4). The effect of flufenamic/mefenamic acid was investigated after a stable cation current was induced by application of 5 mM ATP. DMSO was used as vehicle control in these experiments. Leak-subtracted currents were analysed at +90 mV and data are presented as mean values±s.e.m. For volume-regulated Cl^−^ current recordings, patch electrodes were filled with the following intracellular solution (in mM): N-Methyl-D-Glucamine-Chloride (NMG-Cl), 120; HEPES, 10; EGTA, 11; CaCl_2_, 1; MgCl_2_, 2; Na_2_ATP, 3 (pH 7.3). Cells were kept in iso-osmolar extracellular solution containing (in mM): NMG-Cl, 50; HEPES, 10; D-glucose, 10; CaCl_2_, 2; MgCl_2_, 1; D-mannitol, 170 (300 mosmol per kg, pH 7.3). To activate VRAC currents, superfusion was changed to hypo-osmolar extracellular solution containing no D-mannitol (130 mosmol per kg, pH 7.3). To permit a rapid exchange of solutions for drug application, cells were continuously superfused using a four-barrel microperfusion pipette positioned near the recorded cell. Extracellular solutions containing the indicated NSAID were applied after stable activation of Cl^−^ currents; DMSO was used as vehicle control. All recordings were made at a temperature of 23–26 °C. Whole-cell currents were filtered at 3 kHz and stored for subsequent analyses. Analyses of leak-subtracted currents at +90 mV were performed with the programme FitMaster (HEKA).

### Fluorescence imaging

10^5^ iBMDMs were seeded one day before experiments on glass coverslips in 24-well plates and primed with LPS (1 μg ml^−1^, 4 h) before fluorescence imaging experiments. Subsequently cells were loaded with fluorescent dyes in Na^+^/K^+^ containing extracellular solution at room temperature (20–23 °C): for Ca^2+^ imaging experiments with 3 μM fura-2-acetoxymethylester (Fura-2-AM) for 30 min, for Na^+^ imaging experiments with 10 μM sodium-binding benzofuran-isophthalate acetoxymethyl ester (SBFI-AM; both dyes from Molecular Probes) for 60 min. After washing with extracellular solution, glass coverslips were mounted in a chamber on an inverted Olympus IX50 microscope equipped with a × 40 water immersion objective UApo/340 (Olympus Optical, Co.). The fluorescence imaging system consisted of a monochromator, a charge-coupled device (CCD) camera and a Windows 7 based image processing software (Till Photonics). Cells were exposed to alternating 340±5 and 380±5 nm wavelengths of UV light and emission light was passed through a 400 nm dichroic mirror and a 420 nm long pass emission filter (both Olympus) before image acquisition by the CCD camera. Images were collected every 20 s. Cells were continuously superfused with Na^+^/K^+^-containing extracellular solution using a four-barrel microperfusion pipette positioned in close proximity to the viewing field. Ca^2+^ and Na^+^ influx was induced by extracellular solution containing 5 mM ATP in the presence or absence of mefenamic acid. DMSO was used as vehicle control in all experiments. For each individual cell, mean intensity values from background subtracted pictures were determined and ratios F340/F380 calculated, accordingly. Data are presented as mean values±s.e.m.

### Regulatory volume decrease

THP-1 cells were adjusted to a density of 1 × 10^6^ cells per ml in RPMI media (10% FBS, 1% P/S, 1% Glutamine) and primed with LPS (1 μg ml^−1^, 4 h). A 300 mOsm isotonic buffer was prepared consisting of 147 mM NaCl, 10 mM HEPES, 13 mM glucose, 2 mM KCl, 2 mM CaCl_2_, 1 mM MgCl_2_, adjusted to pH 7.4 using NaOH. From this a 90 mOsm hypotonic buffer was achieved by diluting the isotonic buffer 1:4 with sterile water. Cell size and viability measurements were performed on a BD FACSVerse flow cytometer (BD). THP-1 cells were incubated in a 37 °C water bath and a series of measurements were taken for 60 min with 10,000 events recorded each run. Cell swelling was initiated by aspiration of isotonic buffer followed by addition of hypotonic buffer containing respective drugs. Cell volume measurements by forward scatter width were normalized against the average cell volume before hypotonic stimulus.

### Animal experiments

Animals were maintained under standard laboratory conditions: ambient temperatures of 21 °C (±2 °C), humidity of 40–50%, 12 h light cycle, *ad libitum* access to water and standard rodent chow. All surgeries were performed with the surgeon concealed to the treatment and/or genotype, and all behavioural and histological analyses were performed by a blinded observer. Treatments were randomly allocated. All animal experiments were carried out in accordance with the United Kingdom Animals (Scientific Procedures) Act 1986 and approved by the Home Office and the local Animal Ethical Review Group, University of Manchester.

### Air pouch inflammation model

The air pouch model was used to assess the NLRP3-dependent response to MSU crystals^23^. On day 0 a subcutaneous air pouch was raised in the dorsum of male C57BL/6 mice (30–35 g) by the injection of 4 ml sterile air (filtered through 0.22 μm pore size) with a 25 gauge needle. This was repeated on day 3. On day 7, pouches were injected with 1 ml MSU crystals (3 mg ml^−1^ in sterile saline) or vehicle with or without flufenamic acid (20 mg kg^−1^ in sterile saline, 5% Cremaphor EL, 5% DMSO) or vehicle. Following 6 h incubation, mice were killed by raising the concentration of CO_2_ and pouches were lavaged by injecting 4 ml PBS, 1% BSA, 5 mM EDTA. Lavage was collected from each animal, passed through 100 μm cell strainers before analysis by ELISA as above or by flow cytometry. For the flow cytometry cells were adjusted to a density of 5 × 10^6^ cells per ml in ice-cold PBS, 1% BSA, 5 mM EDTA before plating out on a clear V-bottomed 96-well plate (Thermo Scientific) at 200 μl per well. Cells were stained with antibodies (Anti-CD45 conjugated to FITC at 1 ng ml^−1^, anti-Ly6G conjugated to APC at 1 ng ml^−1^ and anti-F4/80 conjugated to PE at 2.5 ng ml^−1^, all Tonbo Biosciences) for 45 min on ice in the dark before washing twice and fixing in paraformaldehyde (3.7–4.1% in PBS, 200 μl) for 15 min at room temperature. The following day, cells were analysed on a FACSVerse flow cytometer (BD Biosciences) with BD FACSuite software.

### Peritoneal inflammation model

Randomly allocated wild-type (C57BL/6) and NLRP3 KO mice (male, 30–35 g) were dosed i.p. with mefenamic acid (50 mg kg^−1^, 25% (w/v) (2-hydroxy)propyl-β-cyclodextrin in PBS solubilized with 0.1 M NaOH then pH adjusted to 7.4), cyclodextrin vehicle or the selective NLRP3 inhibitor MCC950 (50 mg kg^−1^, PBS) and LPS (1 μg in 0.5 ml, PBS) for 4 h. Following LPS prime, mice were anaesthetized with isoflurane (induced at 3–4% in 33% O_2_, 67% NO_2,_ maintained at 1–2% whilst kept at 37 °C on a heat blanket) before injection with mefenamic acid, MCC950 or vehicle as before and ATP (0.5 ml, 100 mM, PBS) or PBS for 15 min. Peritoneums were lavaged and plasma taken by cardiac puncture before analysis by ELISA for IL-1β and IL-1α.

### Aβ-induced memory deficit

Female Lister Hooded rats initially weighing 200–230 g (*n*=5–10) were used for the following study. Soluble Aβ_1–42_ oligomers were prepared at a concentration of 0.5 mM in sterile 0.1 M phosphate buffer saline as previously described^29^,^43^. Rats were anaesthetized with isoflurane (induction at 4% in O_2_ and maintained at 2% in O_2_) and placed on a stereotaxic frame. Aβ oligomers were injected in the left ventricle (bregma: −0.8 mm anteroposterior, −1.5 mm lateral, −4.5 mm dorsoventral at a rate of 2.5 μl min^−1^; final volume: 10 μl, 5 nmol. Rats underwent surgery (vehicle or Aβ_1–42_ oligomers) on day 0. Rats were treated with daily intraperitoneal (i.p.) injections of vehicle or mefenamic acid (5 mg kg^−1^, 25% (w/v) (2-hydroxy)propyl-β-cyclodextrin in saline solubilized with 0.1 M NaOH then the pH was adjusted to 7.4) from day −1 to day 13. We estimated based on previous literature that the dose of mefenamic acid administered here was likely to be efficacious against the inflammasome^44^,^45^. Treatment was then stopped until the last day of testing (day 35).

### Transgenic mouse model of Alzheimer's disease

Male 3xTgAD mice expressing mutant PS1M146V, APPSwe, TauP301L, and control wild-type (129/C57BL6) mice were originally supplied by Frank LaFerla (Irvine, CA, USA)^46^. At 13–14 months old 3 × TgAD (*n*=20) and wild-type (*n*=20) mice were randomly allocated into mefenamic acid- or vehicle- (60% DMSO, 40% Cremophor A25 Sigma Aldrich) treated groups. The animals were anaesthetized with 2 to 5% isoflurane (30% O_2_ and 70% N_2_O) and implanted with ALZET 200 μl minipump. After four days of priming in the animal, the minipumps reached a sustained administration rate of approximately 25 mg kg^−1^ day^−1^ for 28 days. 36 days after minipump implantation the animals were terminally anaesthetized with 2 to 5% isoflurane (30% O_2_ and 70% N_2_O) and then transcardially perfused with 0.9% saline. The brains were removed and fixed by immersion in 4% paraformaldehyde in 0.2 M phosphate buffer for 24 h followed by a 24 h immersion in 30% sucrose/PBS and then frozen on dry ice. The brains were stored at −80 °C until analysis. One mouse died during minipump implantation.

### Novel object recognition

NOR was performed as previously described^47^,^48^. Briefly, the rats were placed in the NOR box (52 × 52 × 31 cm) and left free to explore two copies of the same object for 3 min (Acquisition phase). Animals were taken out of the box for an inter-trial interval (2 min) then placed back in the same box for a further 3 min, then presented with an identical copy of the previous object and a novel object (Retention phase). Both sessions are recorded on video and the time spent exploring each object was scored. The discrimination index is calculated as (novel−familiar)/(novel+familiar). Rats were tested in the NOR task on day 14 and day 35 after surgery. NOR was also performed using the 3 × TgAD transgenic mice with a white Perspex circular arena 30 cm in diameter, 8 min acquisition phase, 1 h inter-trial interval and a 4 min retention phase. This was performed 18 days after osmotic minipump implantation. Three mice were excluded as they failed to explore for at least 4 s.

### Immunohistochemistry

Free-floating serial 30 μm sections were taken from the wild-type and 3 × TgAD mice using a microtome (Bright Instruments Ltd., UK) and stored in cryoprotectant (30% ethylene glycol, 20% glycerol in 0.2 M phosphate buffer). Sections were washed (3 × ) in PBS for 5 min and blocked for 1 h in 10% normal donkey serum (NDS) in 0.3% Triton X-100 PBS (PBST). This was followed by an overnight incubation with primary antibody in 2% NDS in PBST at 4 °C. Specificity controls were performed on additional 3 × TgAD sections with omission of the primary antibodies. Sections were then washed (3 × ) in PBST and then incubated in with Alexa-488 (visualizing Iba1) and Alexa-594 (visualizing IL-1β) conjugated secondary antibodies (Invitrogen) at 1:500 dilution in 2% NDS in PBST for 2 h. Sections were then washed (3 × ) in PBST and mounted using ProLong Gold Antifade Mountant with DAPI (Thermo Fischer Scientific, Inc., USA). Primary antibodies used were anti-Iba1 (1:1000, Wako Ltd) and anti- IL-1β (1:200, R&D Systems).

### Microscopy and quantification of microglial activation

Images were collected on an Olympus BX51 upright microscope using a × 40 objective and captured using a Coolsnap ES camera. High power field images were taken of the subiculum, CA1 region of the hippocampus and the outer cortex at bregma −2.3 mm, −2.6 mm and −2.9 mm as shown in Supplementary Fig. 10a. These regions were chosen based on previous publications on pathology progression in 3 × TgAD mice^30^. Total microglia were counted and the percentage of IL-1β positive microglia recorded. Microglial morphologies were scored on an activation scale of 0 to 3 based on microglia categories previously described^31^. Scores of 0–1 were considered resting, and 2–3 considered activated (Supplementary Fig. 10b). Each section was treated as a technical replicate, as such scores were averaged for each animal. Counting and scoring were performed by a blinded observer. Example images were collected on a Leica TCS SP5 AOBS upright confocal using a × 63 objective and × 2 confocal zoom. Images were overlaid and stacked using Fiji Image J^49^.

### Statistical analyses

Data are presented as mean values+standard error of the mean (s.e.m). Statistical analyses performed were one-sample *t*-tests, one-way analysis of variance (ANOVA) and two-way ANOVA tests with Sidak corrected *post hoc*. Equal variance and normality were assessed with the Levene's test and the Shapiro–wilk test, respectively, and appropriate transformations were applied when necessary. Accepted levels of significance were *P<0.05, **P<0.01, ***P<0.001. Statistical analyses were carried out using GraphPad Prism or SPSS. Images were processed using Fiji ImageJ^49^ and analysed by manual counting with experimenter blinded to image identity throughout. Flow cytometry data were analysed and populations quantified using FlowJo V10.

### Data availability

The data that support the findings of this study are available from the corresponding author on request.

## Additional information

**How to cite this article:** Daniels, M.J.D. *et al*. Fenamate NSAIDs inhibit the NLRP3 inflammasome and protect against Alzheimer's disease in rodent models. *Nat. Commun.* 7:12504 doi: 10.1038/ncomms12504 (2016).

## Supplementary Material

Supplementary Information Supplementary Figures 1-12.
