# Supplementary material for: Fenamate NSAIDs inhibit the NLRP3 inflammasome and protect against Alzheimer's disease in rodent models
Source: Nat Commun. 2016 Aug 11;7:12504. doi: 10.1038/ncomms12504 (PMC4987536; doi:10.1038/ncomms12504)
Supplement: Supplementary Information — Supplementary Figures 1-12. [file ncomms12504-s1.pdf]

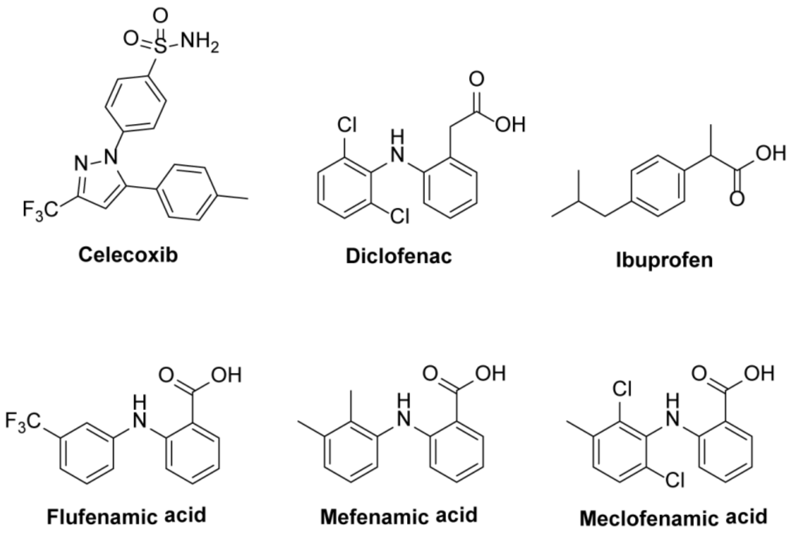

Supplementary Figure 1. Chemical structures of the NSAIDs tested. Non-fenamates are shown in the top row, and fenamates in the row below.

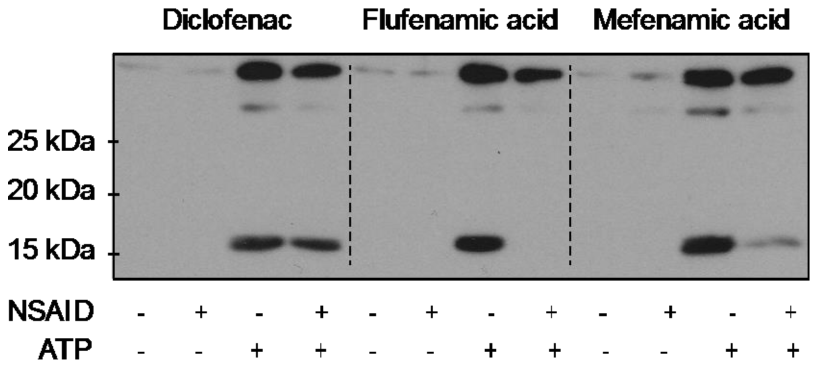

Supplementary Figure 2. The fenamate NSAIDs inhibit processing of IL-1β and caspase-1. (**A**) iBMDMs were primed with LPS (1 µg ml^{-1}, 2 h) treated with NSAID (100 µM, 15 min) and stimulated with ATP (5 mM, 1 h). IL-1β was detected by western blot. Bands indicate pro-IL-1β at 31 kDa and mature IL-1β at 17 kDa.

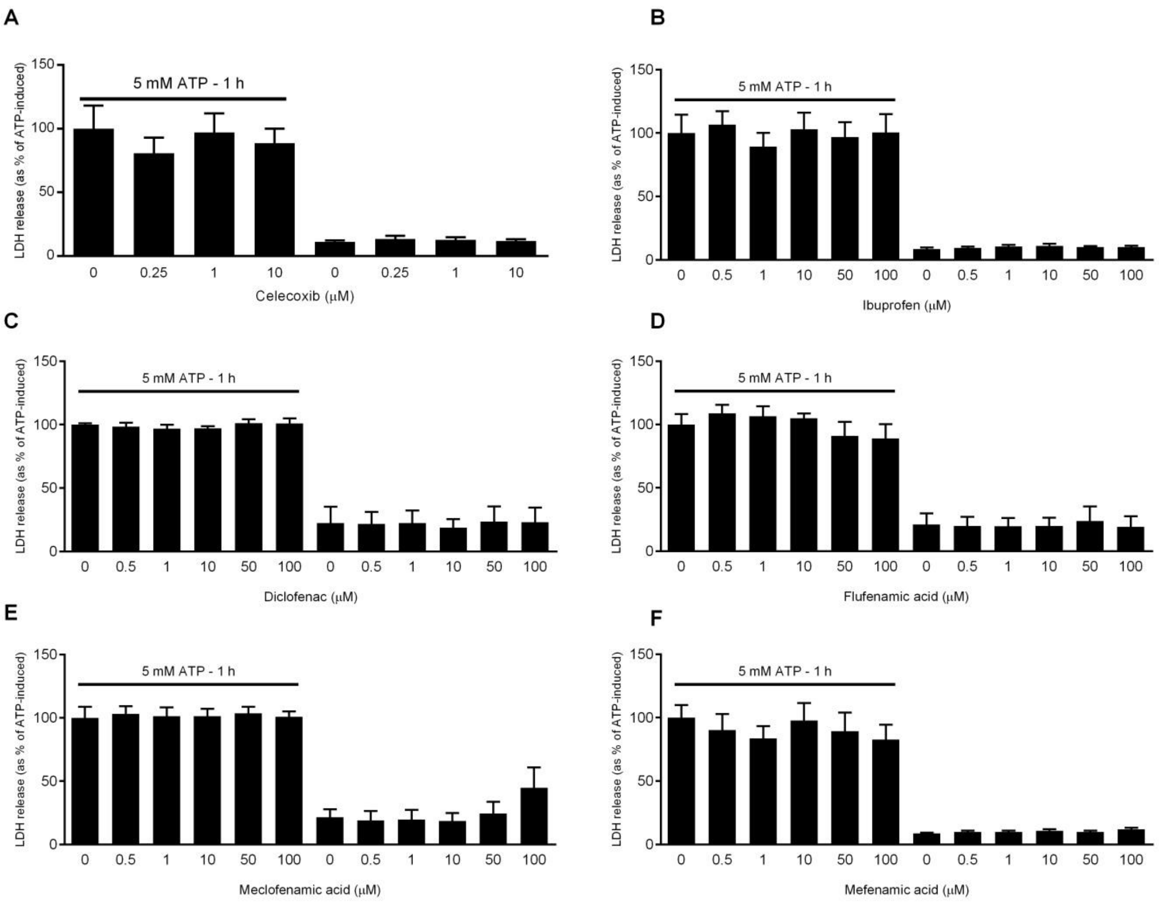

Supplementary Figure 3. None of the NSAIDs tested affect ATP-induced cell death. iBMDMs were primed for 2 h with 1 µg ml^{-1} LPS and pre-treated with (**A**) celecoxib, (**B**) ibuprofen, (**C**) diclofenac, (**D**) flufenamic acid, (**E**) meclofenamic acid or (**F**) mefenamic acid at the indicated concentrations before stimulating with 5 mM ATP for 1 h. Lactate dehydrogenase (LDH) release was evaluated in cell supernatants using a CytoTox assay and data presented as % LDH release compared to ATP alone + s.e.m (n=4)**.** Significance was determined by one-sample t-test vs. hypothetical value of 100 %.

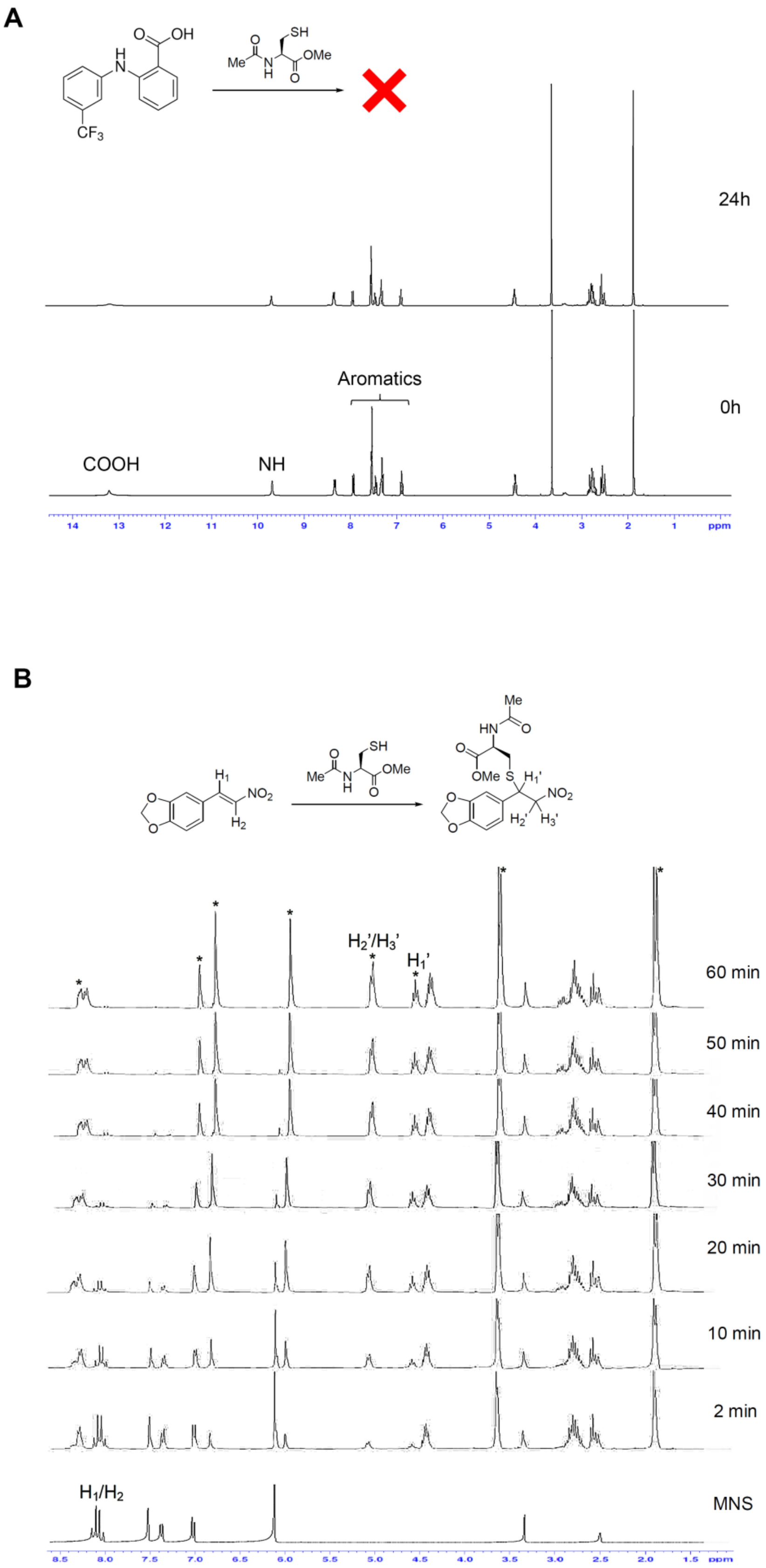

Supplementary Figure 4. Fenamates do not covalently bind cysteine residues. Flufenamic acid or known cysteine modifier MNS (1 eq) and *N*-acetyl-L-cysteine methyl ester (2 eq) were dissolved in DMSO-d_6 (0.5 ml) and kinetically profiled using NMR spectroscopy every 10 min for 2 h and then every 1 h until 24 h.

(**A**) The ^{1}H NMR spectra of flufenamic acid with *N*-acetyl-L-cysteine methyl ester, annotated peaks are for flufenamic acid which remains unchanged after 24 h. (**B**) The ^{1}H NMR spectra for control MNS shows complete conjugation with *N*-acetyl-L-cysteine methyl ester after 24 h. * indicates new peaks associated with cysteine modification, with disappearance of H_1/H_2 doublets of MNS: δ 8.07 and 8.11 (d, *J* = 13.5 Hz, 1H, $C\underline{H}{=}C\underline{H}NO_2$ x 2). These conjugate peaks include: δ 1.846 (s, 3H, $C\underline{H}_3CO$, 1 x diastereoisomer), 1.854 (s, 3H, $C\underline{H}_3CO$, 2 x diastereoisomers, 1:1 ratio), 3.617 (s, 3H, $C\underline{H}_3O$, 1 x diastereoisomer), 3.630 (s, 3H, $C\underline{H}_3O$, 1 x diastereoisomer), 4.60 (t, *J* = 8.0 Hz, 1H, $PhC\underline{H}(S)CH_2NO_2$), 5.03-5.12 (m, 2H, $PhCH(S)C\underline{H}_2NO_2$), 6.02 (s, 2H, $-OC\underline{H}_2O-$), 6.87 (br s, 2H, Ph-$\underline{H}_2$ and Ph-$\underline{H}_5$), 7.05 (s, 1H, Ph-$\underline{H}_6$), 8.40 (d, *J* = 7.8 Hz, N$\underline{H}$, 1 x diastereoisomer), 8.41 (d, *J* = 7.8Hz, N$\underline{H}$, 1 x diastereoisomer).

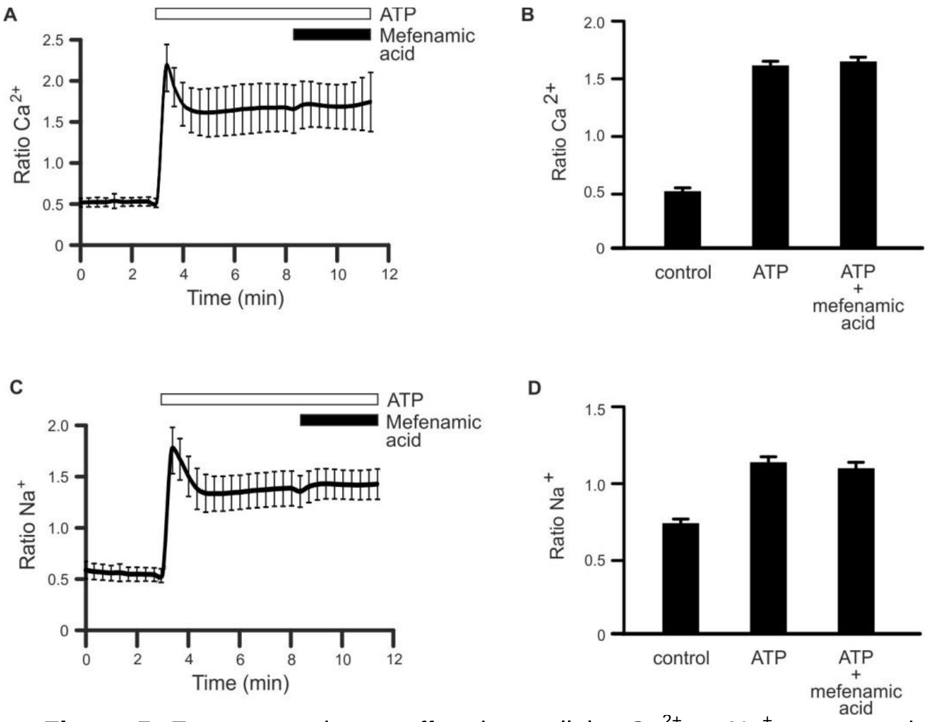

Supplementary Figure 5. Fenamates do not affect intracellular Ca^{2+} or Na^{+} concentrations. iBMDMs were primed with 1 µg ml^{-1} LPS for 4 h. (**A**) Measurements of $[Ca^{2+}]_i$ were performed in Fura 2 loaded cells. 5 mM ATP caused sustained increases in $[Ca^{2+}]_i$, which were unaffected by 100 µM mefenamic acid. Summarised Ca^{2+} signals of 46 individual cells as a function of recording time. (**B**) Steady-state ratiometric Ca^{2+} signals determined before ATP application (control), following application of 5 mM ATP (ATP) and in the presence of ATP and 100 µM mefenamic acid (ATP + mefenamic acid). Data are presented as mean + s.e.m (260 cells in 8 independent experiments). (**C**) Measurements of $[Na^{+}]_i$ were performed in SBFI loaded cells. 5 mM ATP caused sustained increases in $[Na^{+}]_i$, which were unaffected by 100 µM mefenamic acid. Summarised Na^{+} signals of 42 individual cells as a function of recording time. (**D**) Steady state ratiometric Na^{+} signals determined before ATP application (control), following application of 5 mM ATP (ATP) and in the presence of ATP and 100 µM mefenamic acid (ATP + mefenamic acid). Data are presented as mean + s.e.m (277 cells in 8 independent experiments).

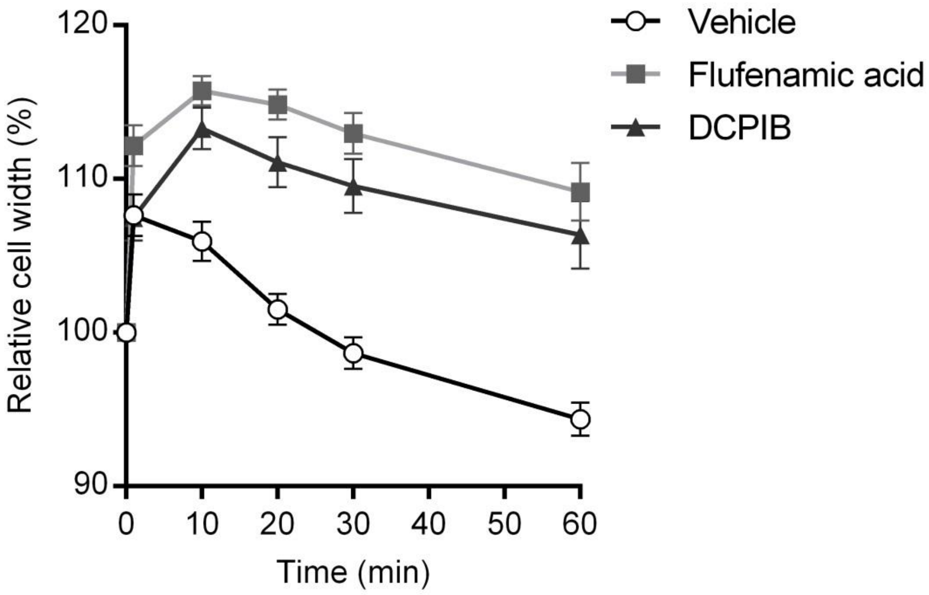

Supplementary Figure 6. Flufenamic acid and DCPIB inhibit the regulatory volume decrease (RVD) in THP-1 cells. THP-1 cells were primed with LPS (1 µg ml^{-1}, 4 h). Cells were then incubated in hypo-osmotic buffer (90 mOsm) plus or minus flufenamic acid (200 µM) or DCPIB (20 µM). Cell size was measured by flow cytometry (forward scatter width). Measurements were taken for 60 mins. Cell volume measurements were normalised against the average cell volume prior to hypotonic stimulus. Data are presented as mean ± s.e.m (n=4).

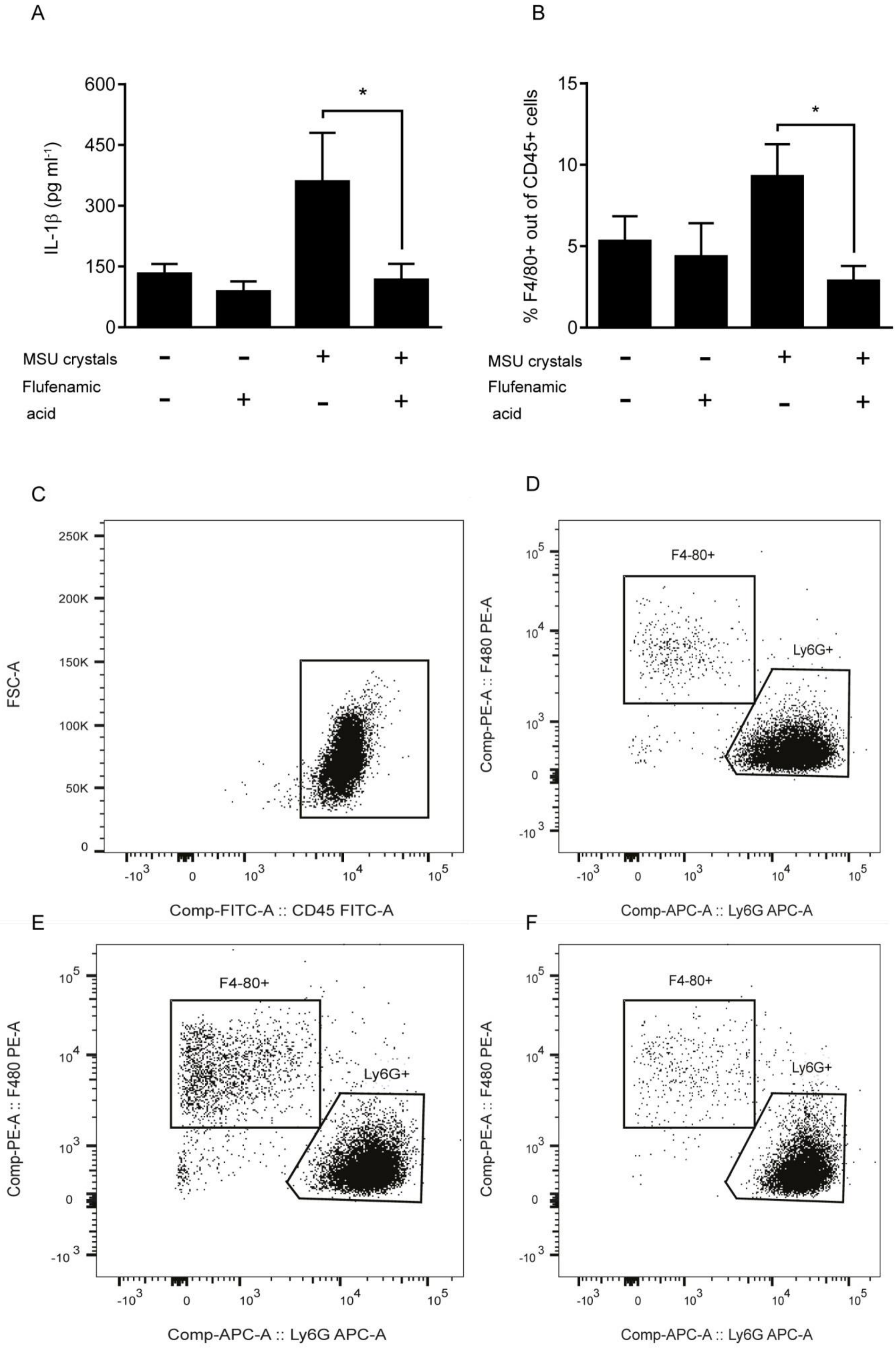

A
B
600
450
300
150
0
IL-1β (pg ml-1)
*
15
10
5
0
% F4/80+ out of CD45+ cells
MSU crystals
Flufenamic acid
C
D
FSC-A
250K
200K
150K
100K
50K
Comp-FITC-A :: CD45 FITC-A
F4-80+
Ly6G+
Comp-PE-A :: F480 PE-A
Comp-APC-A :: Ly6G APC-A
E
F

Supplementary Figure 7. Fenamates inhibit MSU-induced F4/80+ monocyte/ macrophage infiltration and IL-1β production in an air pouch model of inflammation. (**A&B**) Subcutaneous air pouches were raised at the dorsum of C57BL/6 mice before injection of MSU (3 mg ml^{-1}, 6 h) with or without flufenamic acid (20 mg kg^{-1}) or vehicle (5 % Cremaphor EL, 5 % DMSO in sterile saline) in sterile PBS. Pouches were lavaged with 4 ml PBS, 1 % BSA, 5 mM EDTA and analyzed for IL-1β levels by ELISA (**A**) or leukocyte populations by flow cytometry (**B**). Upon quantification of total cell number it was observed that addition of 20 mg kg^{-1} flufenamic acid significantly reduced MSU-induced total F4/80+ macrophage infiltration from 1.3 ± 0.61 to 0.14 ± 0.026 million cells (mean ± s.e.m, P<0.01, data not shown). (**C-F**) Immune cells were analyzed by gating for CD45+ leukocytes (**C**) then Ly6G+, F4/80- neutrophils or Ly6G-, F4/80+ macrophages/monocytes (**D-F**). Representative examples are shown of cell populations from mice treated with vehicle then vehicle (**D**), vehicle then MSU (**E**) and flufenamic acid then MSU (**F**). n=4-6. ELISA data are presented as mean IL-1β detected in lavage + s.e.m. Flow cytometry data are presented as mean % F4/80+, Ly6G- population out of CD45+ population + s.e.m. *P<0.05 determined by one-way ANOVA and Sidak corrected planned contrast post-hoc test.

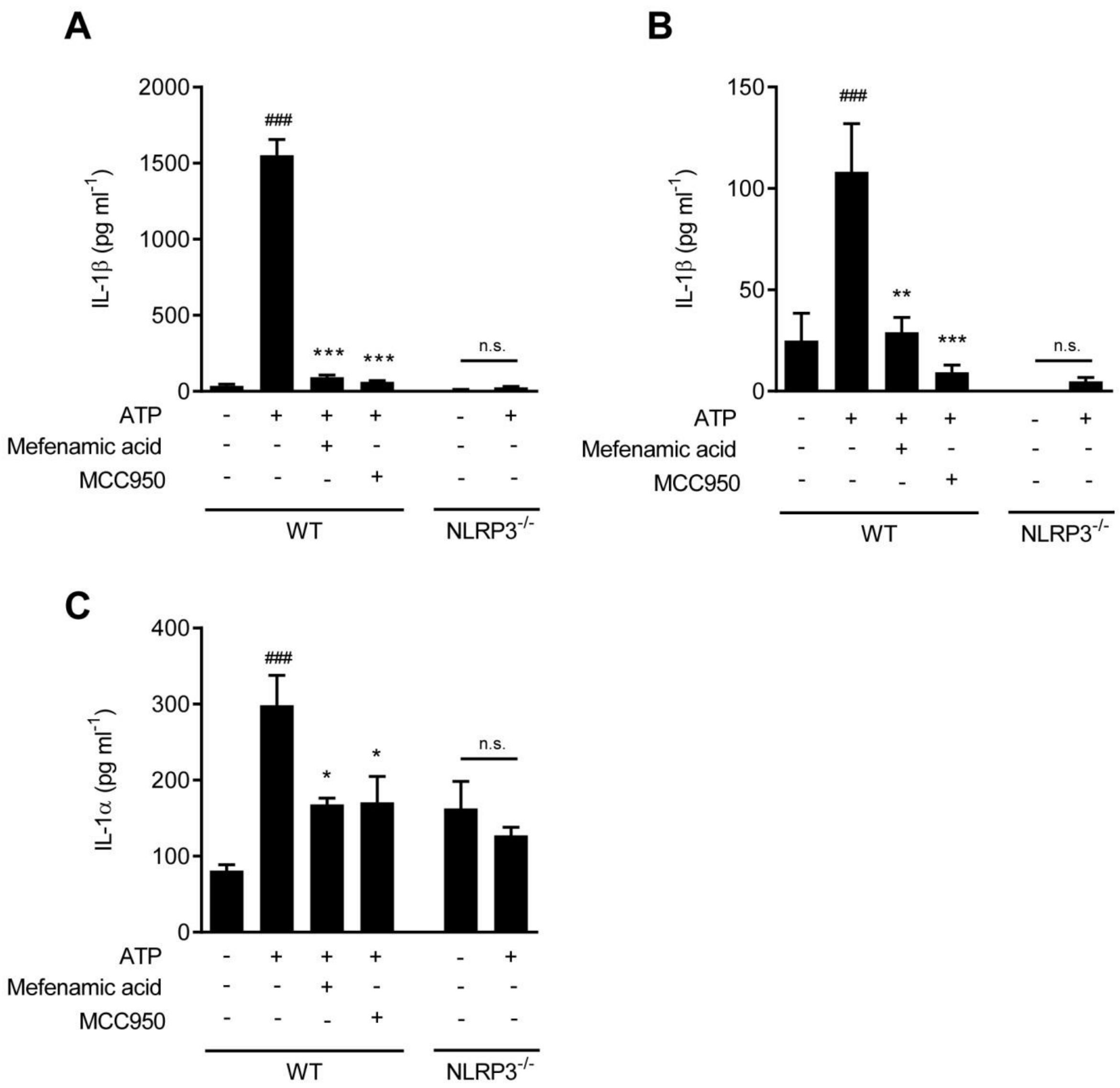

Supplementary Figure 8. Mefenamic acid inhibits ATP-induced IL-1 production in a peritoneal model of NLRP3-dependent inflammation. Wild-type (WT) and NLRP3$^{-/-}$ mice were pretreated intraperitoneally (i.p.) with 50 mg kg^{-1} mefenamic acid, the specific NLRP3 inhibitor MCC950, or vehicle before priming with 1 µg LPS. 4 h following the LPS injection animals were injected with drugs before stimulation with 100 mM ATP i.p. for 15 mins under anesthesia. IL-1β levels in peritoneal lavage (**A**) and plasma (**B**), and IL-1α levels in lavage (**C**) were measured by ELISA. Data are presented as mean IL-1 levels + s.e.m (n=3-4). ###$p<0.001$ compared to WT saline control group; *$p<0.05$, **$p<0.01$, ***$p<0.001$ compared to WT ATP-treated groups determined by one-way ANOVA with Sidak’s multiple comparisons.

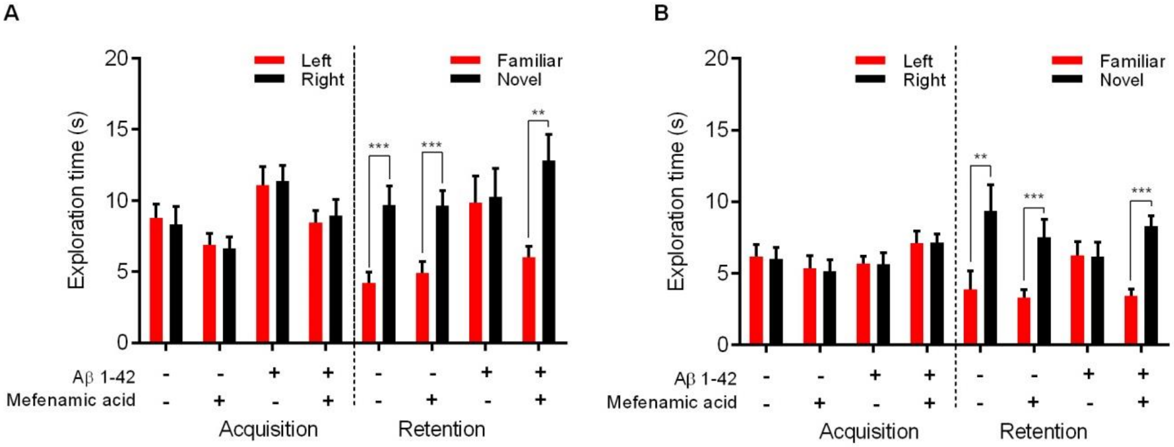

Supplementary Figure 9. Mefenamic acid reverses Aβ1-42 induced deficits in novel object recognition *in vivo*. Acute unilateral intracerebroventricular injection of soluble Aβ1-42 on day 0 (5 nmol in 10 µl) was followed by 14 days (starting one day prior to surgery) of i.p. injection of mefenamic acid (5 mg kg^{-1}) or vehicle. Animals were then tested in the NOR task on day 14 and day 35 post-surgery. Exploration of the objects at 14 d (**A**) and 35 d (**B**) post-surgery are presented as mean exploration time + s.e.m (n=5-10 per group). **p<0.01 ***p<0.001 Familiar versus Novel, determined by one-way ANOVA followed by a within group paired student t-tests.

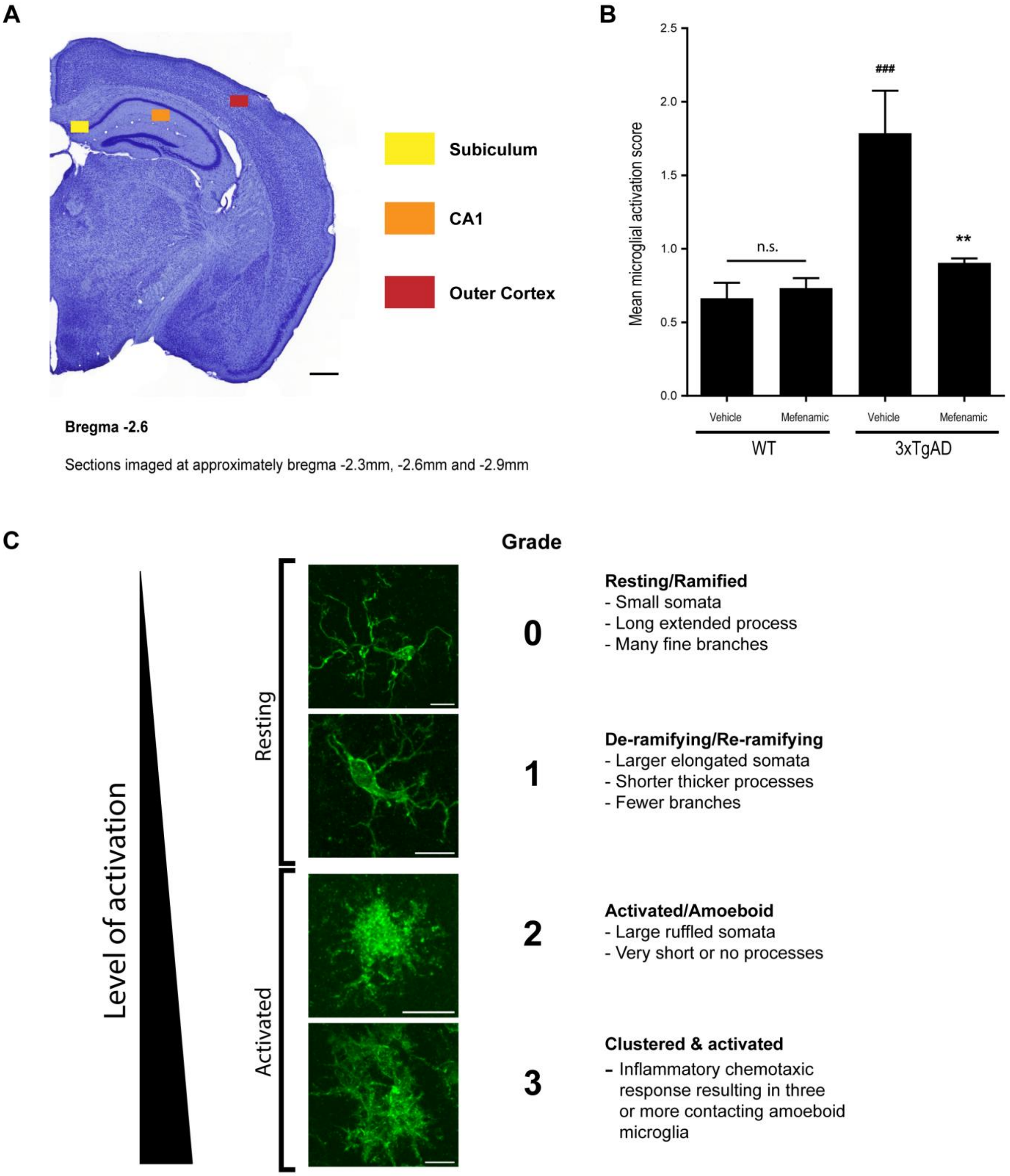

Supplementary Figure 10. (**A**) Locations of high-power field images depicted on a transverse section of mouse brain stained with cresyl violet. Images were taken as near as possible to the locations indicated at approximately bregma -2.3 mm, -2.6 mm and -2.9 mm. Scale bar is 50 µm. (**B**) Mean activation score of Iba1 stained microglia within the subicula of 3xTgAD and WT mice following vehicle or mefenamic acid treatment. Data are presented as mean + s.e.m (n=8-10). Statistical analyses performed using two-way ANOVA followed by Sidak corrected post-hoc analysis. ###p<0.001 compared to vehicle/WT animals and **p<0.01 compared to vehicle/3xTgAD mice. (**C**) Method of rating the activation state of microglia on a scale of 0-3 and the method of binary identification of activated or resting microglia. Scale bars are 10 µm.

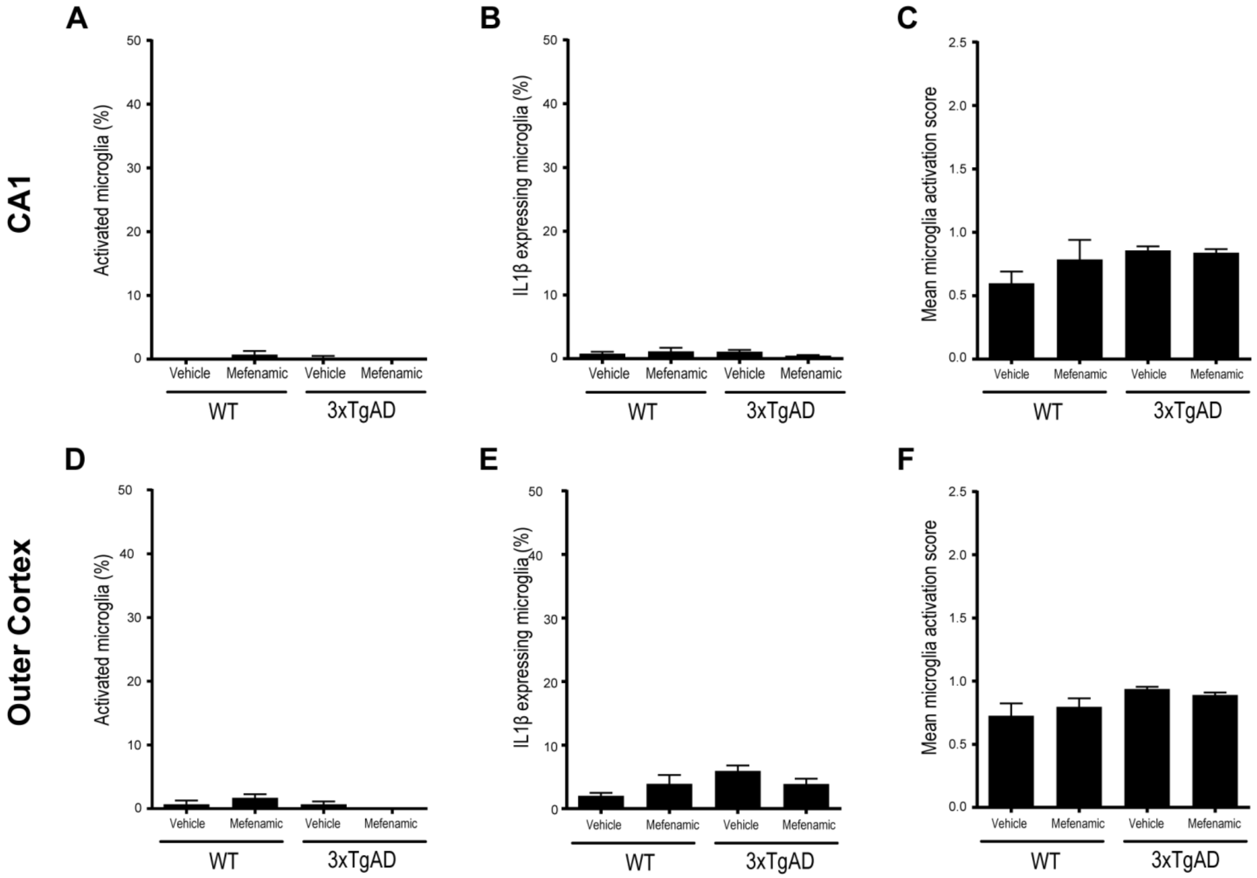

Supplementary Figure 11. There were no significant changes in microglia morphology or IL-1β expression in the CA1 (**A-C**) or outer cortical (**D-F**) regions in the triple transgenic Alzheimer's mouse model 3xTgAD. (**A&D**) Percentage of activated microglia in the CA1 region (**A**) and outer cortex (**D**). (**B&E**) Percentage of IL-1β expressing microglia in the CA1 regions (**B**) and outer cortex (**E**). (**C&F**) Mean microglial activation score of microglia in the CA1 regions (**C**) and outer cortex (**F**). Data are presented as mean + s.e.m (n=8-10). Statistical analyses performed using two-way ANOVA and no significant effects were observed.

A

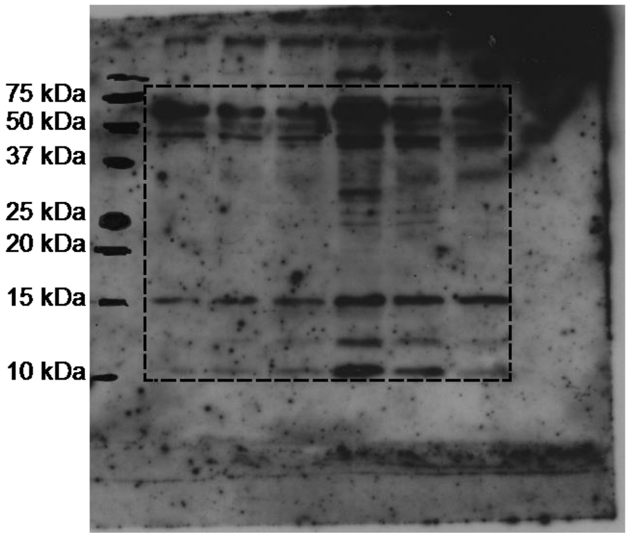

B

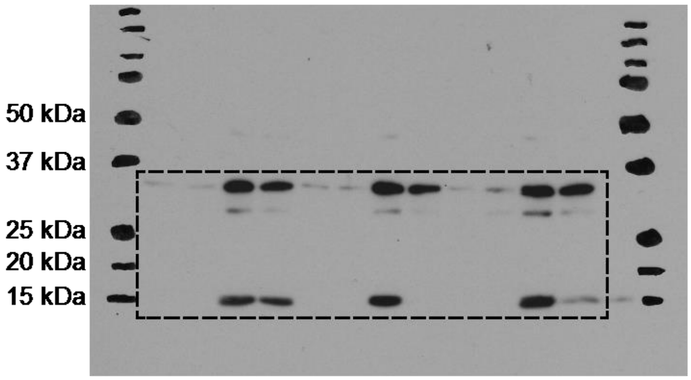

Supplementary Figure 12. Uncropped blots appearing in Figure 2C (**A**) and Supplementary Figure 2 (**B**).
